# Editorial: Brain function alterations in populations with musculoskeletal disorders: now let’s talk about it

**DOI:** 10.3389/fspor.2026.1913466

**Published:** 2026-07-16

**Authors:** Xiao’ao Xue, Christopher J. Burcal, Bruno Tassignon, Alan R. Needle

**Affiliations:** 1 Department of Sports Medicine, Huashan Hospital, Fudan University, Shanghai, China; 2 Department of Rehabilitation, Jiangwan Hospital of Hongkou District, Shanghai University of Medicine and Health Sciences, Shanghai, China; 3 School of Health and Kinesiology, University of Nebraska Omaha, Omaha, NE, United States; 4 Faculty of Rehabilitation Sciences, REVAL Rehabilitation Research Center, Hasselt University, Diepenbeek, Belgium; 5 Department of Kinesiology, Appalachian State University, Boone, NC, United States; 6 Department of Rehabilitation Sciences, Appalachian State University, Boone, NC, United States

**Keywords:** anterior cruciate ligament injury, chronic ankle instability, musculoskeletal injury, neurocognition, neuroplasticity, postural control, return to sport

Musculoskeletal injuries have traditionally been managed as local tissue problems (e.g., a ruptured ligament, a painful tendon). Yet peripheral healing alone does not always explain clinical recovery. Many patients and athletes regain range of motion, strength, and basic functional capacity, but continue to report instability, fail to return to their previous level of activity or sport, or sustain recurrent injury (1). These observations have encouraged a broader view of musculoskeletal disorders, in which altered afferent input, sensorimotor integration, allocation of cognitive resources, and brain neuroplasticity may contribute to persistent dysfunction (2–4).

This Research Topic, “*Brain Function Alterations in Populations with Musculoskeletal Disorders: Now let's talk about it*”, was developed to highlight the central nervous system dimension of musculoskeletal injury and rehabilitation. The four contributing articles span knee and ankle ligamentous injury, sensory-cognitive assessment, return-to-sport (RTS) decision-making, and cerebellar-targeted neuromodulation. Although diverse in methodology and scope, they converge on a common question: to what extent do brain-related adaptations influence recovery after musculoskeletal injury, and how should these adaptations be considered in rehabilitation and RTS practice?

One challenge in this domain is establishing whether central alterations persist throughout recovery. Addressing this question, Jiménez-Martínez et al. broadened the discussion of recovery by examining cognitive performance in open-skill sport athletes with anterior cruciate ligament (ACL) injury from the preoperative period through the postoperative rehabilitation and eventual RTS. Using the Flanker (inhibitory control) and Multiple Object Tracking (attention) tasks, the authors observed that cognitive performance improved after ACL reconstruction, but persistent cognitive deficits remained compared to athletes without ACL injury at the completion of rehabilitation. These findings suggest that recovery of physical function may not coincide with the recovery of perceptual-cognitive capacities required for returning to open-skill sports and their demands. For athletes participating in open-skill sports, where visual tracking, response inhibition, and rapid decision-making are fundamental performance requirements, this study advocates that rehabilitation and medical clearance should encompass recovery of the perceptual-cognitive capacities required for safe RTS (4, 5).

While the ACL study highlights the persistence of cognitive alterations following injury and rehabilitation, two complementary contributions by Picot et al. help place these findings within a broader conceptual framework for lateral ankle sprain (LAS) and chronic ankle instability (CAI). In part 1, the authors briefly review current evidence regarding cognitive impairments and sensory reweighting in LAS and CAI. Their synthesis indicates that persistent symptoms after LAS should not be attributed solely to mechanical instability or local sensorimotor deficits (3, 6). The authors make a case for cognitive impairments and sensory reweighting as clinically relevant, yet often overlooked, dimensions of CAI, including altered visual reliance, attentional resource allocation, cognitive-motor integration, processing speed, and inhibitory control (7, 8). This perspective is particularly relevant to clinicians because RTS decisions after LAS are still often based on time, symptoms, or isolated functional tests, whereas sport participation requires athletes to regulate movement while processing visual and cognitive demands (9). By framing CAI as a condition involving adaptations across the sensorimotor system, this work contributes to a growing recognition that musculoskeletal injuries often have consequences extending beyond the injured tissue itself.

Part 2 translates the theory to a practical RTS application. Building on the Ankle-GO™ score, Picot et al. proposed a “β(rain)” extension that integrates dual-task paradigms and visual-sensory constraints into this RTS assessment (10). Thus, similarly to the contribution of Jiménez-Martínez et al., the authors advocate that RTS evaluation should examine conditions that better reflect the perceptual, cognitive, and motor demands encountered during sport participation. This advanced framework represents an important attempt to translate emerging knowledge regarding brain-related adaptations into clinically meaningful assessment strategies. Although proposed for LAS and CAI, this principle may also inform rehabilitation after other musculoskeletal injuries in which sensory-cognitive demands influence a safe return to activity.

The final contribution by Zheng et al. extended the Research Topic further by addressing a potential novel intervention. In a randomized, sham-controlled laboratory study, the authors researched whether cerebellar transcranial direct current stimulation could modify postural control and resting-state brain activity in individuals with CAI. Building on previous work linking CAI-related proprioception deficits to cerebellar vermis alterations (11), the authors found that a single 20-min stimulation session improved specific laboratory measures of postural control and was associated with altered cerebellar functional coherence. However, the Balance Error Scoring System scores did not show clear between-group effects. These findings suggest that neural mechanisms associated with musculoskeletal dysfunction may represent modifiable therapeutic targets. As such, the study provides an early example of how insights from neuroscience may eventually inform novel rehabilitation approaches (12).

Several priorities for future research emerge from this Research Topic. Longitudinal studies are needed to clarify the causal relationship between injury occurrence, neural adaptation and clinical outcomes. Candidate neurocognitive, sensory reweighting, and neurophysiologic markers should be evaluated against common clinical measures, including balance, and endpoints including recurrent injury, delayed recovery, RTS success, and long-term function. Assessment paradigms should evolve beyond isolated clinical tasks toward conditions reflecting reflect real-world cognitive, visual, and motor demands. Lastly, clinical trials should determine whether cognitive-motor training, sensory-motor targeted rehabilitation, or neural modulation can improve clinically relevant outcomes, such as patient-reported function, sport performance, reinjury risk, and long-term participation, clarifying intervention dosing, long-term retention of effects (>6 months), and injury rates, rather than relying solely on laboratory-based outcomes.

Collectively, the contributions in this Research Topic reflect a progression increasingly shaping musculoskeletal rehabilitation research: from documenting persistent cognitive alterations following injury, to understanding the sensory and cognitive mechanisms that may underlie persistent dysfunction, to incorporating these concepts into clinically meaningful assessment frameworks, and finally to exploring whether neural adaptations themselves can be targeted therapeutically. Together, these studies support a more integrated view of musculoskeletal disorders in which tissue healing, sensorimotor function, cognition, and neuroplasticity are considered interacting components of recovery. By using multidisciplinary perspectives from neuroscience, biomechanics, psychology, rehabilitation and sports medicine, this Research Topic highlights the value of moving beyond the peripheral focus on injury and recovery and encourages continued investigation into the role of brain-related mechanisms in musculoskeletal health. That conversation has begun; now, it should continue.
